# Predicting electrocardiogram interpretation performance in Advanced Cardiovascular Life Support simulation: comparing knowledge tests and simulation performance among Mexican medical students

**DOI:** 10.7717/peerj.6632

**Published:** 2019-03-15

**Authors:** Michael William Smith, David Abarca Rondero

**Affiliations:** 1 Department of Industrial and Mechanical Engineering, Universidad de las Américas Puebla, San Andres Cholula, Puebla, Mexico; 2 Department of Health Sciences, Universidad de las Américas Puebla, San Andres Cholula, Puebla, Mexico

**Keywords:** Simulation training, Educational measurement, Electrocardiography, Advanced Cardiac Life Support, Knowledge

## Abstract

**Background:**

Simulation plays a key role in assessing performance in Advanced Cardiovascular Life Support (ACLS). Traditional knowledge tests are also important for assessing the cognitive elements of ACLS performance. However, the association between the two has not been established. In this study, we focus on one important element in ACLS—interpretation of electrocardiograms (ECG)—and the potential of knowledge tests to serve as predictors of improvement in ACLS performance.

**Methods:**

We looked at the correlation between Mexican medical students’ improvement in ECG interpretation performance in ACLS megacode simulations (from the start of the semester to the end of the semester), and their scores on ECG interpretation knowledge tests.

**Results:**

We found significant improvement in ECG interpretation in ACLS megacode simulation (from pre-semester to post-semester), but this was not predicted by the ECG interpretation knowledge test scores. The correlation was .079 (*p* = 0.66).

**Conclusions:**

These results suggest that even cognitive tasks such as ECG interpretation can be expressed and assessed differently in simulation versus traditional knowledge testing.

## Introduction

The ability to resuscitate a patient is the epitome of a life-or-death skill. Assessment of the knowledge and skills of Advanced Cardiovascular Life Support (ACLS) is an important way to determine competency in this ability (Ringsted et al., 2007). Patient simulators make it possible to assess ACLS resuscitation ability in a reliable and consistent manner (Scalese, Obeso & Issenberg, 2008).

There are limitations to this use of simulation, and reasons to explore additional assessment techniques. In representing a single specific case, a simulation scenario provides a valid analog to the actual situation of a patient in cardiac arrest. But this means that the provider is assessed only on one of the many possible types of cases. Another limitation is the many costs associated with the use of medical simulators, including the cost of the simulator and other equipment, the personnel, and the facility (Zendejas et al., 2013).

It has been suggested that knowledge tests could serve as an alternative to assessment via ACLS simulation (Chamberlain et al., 2003). However, this is not supported by overviews of the studies comparing performance in knowledge tests and ACLS simulation (Mancini et al., 2010; Bhanji et al., 2015).

Some studies have found moderate correlations between written test performance and simulation performance. Strom et al. (2015) compared the multiple-choice test performance and the simulation scenario performance of 19 US medical students. The correlation was 0.48 (*p* = 0.04). Napier et al. (2009) also looked at multiple choice test performance and simulation scenario performance, with 537 participants (mostly doctors and nurses). The correlation was 0.336 (*p* < 0.01).

Other studies have failed to find correlations between written tests and simulation performance. Rodgers, Bhanji & McKee (2010) worked with 34 nursing students to test the relationship between knowledge test results and megacode simulation performance (a type of complex ACLS scenario developed for assessment; Kaye & Mancini, 1986). The correlation was 0.194 (*p* = 0.272). Roh & Issenberg (2014) looked at 124 nursing students doing emergency department clinical rotations. They compared results of multiple-choice question on CPR (specifically on knowledge of compression and knowledge of ventilation) with deficits in psychomotor performance doing CPR on a manikin (collecting data on compression and ventilation performance). The correlation for compression was −0.06 (*p* = 0.510); the correlation for ventilation was −0.103 *p* = 0.257).

With the exception of Roh & Issenberg (2014), these studies analyzed knowledge test results and simulation performance scores in the aggregate, without considering specific elements by themselves. This has been noted as a limitation (Strom et al., 2015). The different components of ACLS simulation performance may not be equally accessible to assessment or prediction via written knowledge test questions. Some components involve primarily psychomotor skills or social interaction; others are more cognitive.

The interpretation of electrocardiograms (ECG) is a task that relies heavily on cognitive abilities (Salerno, Alguire & Waxman, 2003; Kodra et al., 2016; Viljoen et al., 2017). As such, we suspect that knowledge testing of ECG interpretation may be a sufficiently close analog of ECG interpretation in ACLS simulation.

Knowledge tests of ECG interpretation might serve as predictors for performance on the ECG interpretation tasks in ACLS simulation. Establishing this potential would support the use of knowledge tests of ECG interpretation as efficient screening assessments to be used in preparation for ACLS simulations. In this manner, the utility of the simulation activity would be less at risk of incorrect ECG interpretation. The simulation would be able to focus more on tasks that are dependent on the simulator environment, such as psychomotor or social interaction tasks. In this study, we test the hypothesis that knowledge tests of ECG interpretation will predict performance of ECG interpretation occurring in megacode simulation.

## Methods

**Figure 1:**
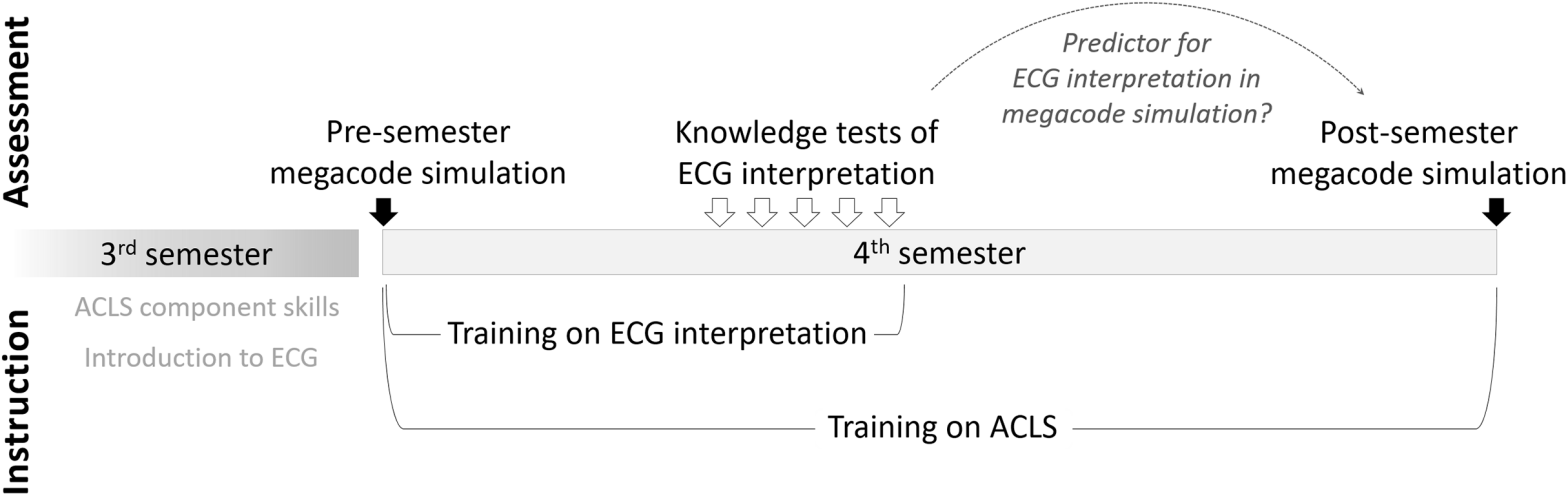
Overview of instructional and assessment activities.

In this study, we compared the performance of medical students on knowledge tests of ECG interpretation with their performance at ECG interpretation in a megacode simulation at the end of the semester, in order to see if the knowledge tests predict ECG interpretation performance in the simulation. We also collected baseline data via a megacode simulation at the beginning of the semester (see Fig. 1).

### Setting and participants

We conducted this study at Universidad de las Americas Puebla (UDLAP), Mexico. Inclusion criteria was enrollment in the fourth semester Medical Simulation Laboratory course for the Autumn 2016 or Spring 2017 semesters. It is a required course in the Medical Surgeon 5-year bachelor’s degree program.

Participation in the assessment and instructional activities was mandatory for the enrolled students, but permission for the researchers to use each student’s data was voluntary. No student declined. Approval for this study was obtained from the UDLAP Department of Health Sciences’ Ethics Board. Verbal informed consent was obtained. A total of 42 students participated (34 women, eight men; 14 during Autumn 2016, 28 during Spring 2017). The average age was 20.5.

The fourth semester Medical Simulation Laboratory course was focused on advanced life support and trauma care. It continued on the electrocardiography training and life support component skills that the students started in the third semester. The training on ACLS included AHA protocols, but was done as part of this 16 week long medical school course and not an AHA short course. ACLS instructional content included: assessing vital signs via multi-parametric monitors; using defibrillators; understanding and following AHA ACLS algorithms; assessing and managing team dynamics; and resuscitation activities on medium- and high-fidelity simulators (including the Laerdal SimMan 3G manikins used in the megacode simulations). It included frequent activities with high-fidelity simulators and debriefing sessions.

### Megacode simulation protocol

At the beginning of the semester, we measured baseline performance in a megacode simulation. Each student performed as team leader during a particular megacode scenario. The specific presentation of the scenario varied across the students, but the same general algorithm always applied (American Heart Association, 2015). The general pattern across the different scenarios was that the patient first presented with an abnormal pulse rate, degrading into abnormal ventricular activity and eventually to a lack of pulse. With appropriate interventions, there would be a return of spontaneous circulation. The scenario lasted approximately 8 min. We used various scenarios across the students because this enables better evaluation of the course at enabling ACLS over various types of cases.

Two trained observers (one faculty member, one medical graduate) observed the live performance and evaluated performance using a 23 item AHA megacode scoring rubric (which included some items specifically about ECG interpretation) (AHA, 2011). Immediately afterwards the student and another team member (of the student’s choosing) critically reviewed the observers’ scores. The multi-camera audio–video recording was used to resolve disagreements over the assessment.

This was repeated at the end of the semester, using the same specific presentation that the student encountered at the beginning of the semester. We repeated each student’s specific scenario in order to make a more accurate pre vs. post comparison. In between the pre-semester and post-semester megacode simulations were 16 weeks of full-time medical school training.

### ECG interpretation: instruction and assessment

Part of the fourth semester Medical Simulation Laboratory course was instruction in ECG use and interpretation. Instructional content included: principles of ECG and skills in its use; reading static waveforms; and reading dynamic waveforms on a monitor. ECG interpretation was covered primarily during weeks 2–5 of the 16-week semester, during which the students were given multiple-choice tests on interpretation of particular waves. Different rhythm types were covered and tested each week. Among the multiple-choice questions were five specific questions about identification of ECG waves of the same type as those encountered in the megacode simulation (depending on the specific presentation): Sinus bradycardia, supraventricular tachycardia, ventricular tachycardia, ventricular fibrillation, and asystole.

### Data analysis

The outcome data consisted of:

- Pre-semester and post-semester performance on the megacode simulation (overall, and the average of the components specific to ECG interpretation—see Table 1); and
- Knowledge test scores on ECG interpretation (five questions on interpretation of specific ECG waves related to the megacode simulation—see Table 2).

**Table 1:** Improvements in megacode simulation scores from pre-semester to post-semester.

Megacode simulation	*N*	Pre-semester		Post-semester		*p*-Value
		Mean	SD	Mean	SD	
Overall performance	42	0.08	0.069	0.62	0.126	<0.001
ECG interpretation	42	0.00	0.00	0.71	0.296	<0.001

**Table 2:** Results of knowledge tests for ECG interpretation.

ECG wave type	*N*	Mean	SD
Sinus bradycardia	38	0.61	0.49
Supraventricular tachycardia	40	0.65	0.48
Ventricular tachycardia	38	0.32	0.46
Ventricular fibrillation	40	0.65	0.48
Asystole	38	0.74	0.44

We used paired *t*-tests to compare pre-semester and post-semester megacode performance (overall, and specific to ECG interpretation). Using Pearson’s R, we analyzed the correlation between the post-semester ECG-related megacode performance and the knowledge tests for ECG interpretation (averaging across the five questions). SPSS was used to perform the statistical tests.

## Results

The results of the paired *t*-tests show that performance on the megacode simulation significantly improved from pre to post. This includes overall performance (which improved from 8% correct to 62% correct, *p* < 0.001), and performance specific to ECG interpretation (0–71%, *p* < 0.001). See Table 1. These results show that initial simulation performance was very poor (despite prior training), especially regarding the ECG interpretation aspects of the megacode, which was zero. Thus, for our results the post-semester score for ECG interpretation in the simulation is the same as the improvement from pre-semester to post-semester. These results also show that the improvements in ability to interpret ECGs during a simulation (which we have hypothesized will be predicted by the ECG knowledge tests) were sizeable and significant.

The scores from the knowledge test questions on ECG interpretation of five different wave types are shown in Table 2. Overall students were 59% correct (worse at ventricular tachycardia, better at asystole).

The Pearson’s R correlation between the overall % correct on the knowledge test ECG interpretations and the post-semester score of ECG interpretation in the megacode simulation was 0.079 (*p* = 0.66). Even though there was variability in the ECG interpretation in the post-semester megacode, and variability in the knowledge test ECG interpretation, these were not correlated. The results do not support our hypothesis that ECG knowledge tests can be a predictor for ECG interpretation in simulation.

## Discussion

We measured students’ megacode simulation performance at the beginning and end of their fourth semester of undergraduate medical school. We compared pre-semester and post-semester performance on the megacode simulation (overall performance, and performance just on components involving ECG interpretation). We looked for correlations between their post-semester performance of elements in the megacode simulation related to ECG interpretation, and knowledge tests of ECG interpretation conducted during the semester.

We found significant improvement from pre-semester to post-semester in both the overall and the ECG interpretation aspects of the megacode simulation. This is an unsurprising finding, as ACLS competency is responsive to training (Greif et al., 2015; Bhanji et al., 2015), and simulation performance is sensitive to those training-based improvements in competency (Perkins, 2007).

We found no evidence that associates this improvement with their ECG interpretation knowledge that was assessed via multiple choice questions in the middle of the semester. There still may be quick and inexpensive ways to predict performance at ECG interpretation in simulations, but multiple-choice questions is not one of them.

The intervening time between the ECG knowledge tests and the post-semester megacode performance may have contributed to these findings. We do not have indicators of continued learning of ECG interpretation on the part of the students during the second half of the semester, but there is every reason to suspect that students continued to enhance their ECG interpretation abilities. The post-semester megacode scores on ECG-related tasks are higher than would be expected based on students’ performance on the ECG knowledge tests several weeks prior.

At a more fundamental level, knowledge tests and simulation performance measure different things. Performance in a simulation involves not just knowledge but its application in context (Strom et al., 2015). Compared to a knowledge test, the expression of knowledge in a simulation is affected by contextual factors (the team, the manikin, and the lab environment) and scored by observers relying on explicit behavior, not mental content (Boulet et al., 2011). This study supports these viewpoints.

Knowledge tests are still useful tools, of course. However, it does not appear that they can function as substitutes (whole or in part, e.g., for ECG interpretation) for simulation assessment in its roles of checking decay of unused ACLS skills (Yang et al., 2012), high-stakes testing (McGaghie et al., 2010), or evaluating the impact of ACLS training (Mancini et al., 2010; Bhanji et al., 2015).

## Limitations

One limitation is the sample size. A total of 42 students participated, and complete data was available for only 38. A much larger sample size would have been more likely to detect significant relationships.

As mentioned above, the duration between the knowledge tests and the post-semester megacode may have contributed to the lack of correlation. A test of concurrent validity (comparing knowledge test and simulation performance at roughly the same time) would presumably show a stronger correlation than our test of predictive validity.

Another caveat is that we compared aggregate ECG knowledge test performance to aggregate ECG interpretation performance in the simulator. We did not compare interpretation of one specific wave in a written test to interpretation of that same wave in the megacode simulation.

It is possible that with comparing the knowledge test ECG interpretations and the megacode simulation interpretations at the same time, and by focusing on how a knowledge test on one specific wave correlates with interpretation of that same wave in a simulation, we would obtain higher correlation values. However, the practical value of establishing concurrent validity for very specific assessment is questionable. After all, the goal of assessment for ACLS is to ensure competent delivery for a wide range of cases.

## Conclusions

Contrary to our hypothesis, the pre-semester to post-semester improvement in ECG interpretation during megacode simulation was not predicted by ECG interpretation knowledge tests during the semester. This could be due to continued skill enhancement between the knowledge tests and the post-semester simulation, and/or contextual factors present in the megacode simulation. An implication of these results is that knowledge testing to assess performance of cognitive tasks (such as ECG interpretation) is important and useful (and may help assess necessary knowledge), but is unlikely to be sufficient to establish competency for that task in realistic, dynamic situations.

## Supplemental Information

10.7717/peerj.6632/supp-1 Supplemental Information 1 Raw data: Data ECG_tests ACLS_pre ALCS_post. Rows list each participant and columns list the variables. The ECG knowledge tests and the pre- and post-ACLS tasks (0 for wrong/unsuccessful, 1 for correct/successful). Rows 1 and 2 contain identifying headers.

## Additional Information and Declarations

### Data Availability

The following information was supplied regarding data availability: Raw data is available as a Supplemental File.
